# ﻿Redescription of *Tylosmaindroni* Giordani Soika, 1954 (Crustacea, Isopoda, Oniscidea) based on SEM and molecular data

**DOI:** 10.3897/zookeys.1087.76668

**Published:** 2022-02-23

**Authors:** Valiallah Khalaji-Pirbalouty, Hamzeh Oraie, Carlos A. Santamaria, Johann Wolfgang Wägele

**Affiliations:** 1 Department of Biology, Faculty of Basic Science, Shahrekord University, Shahrekord, Iran Shahrekord University Shahrekord Iran; 2 Department of Biology, University of Tampa, 401 W Kennedy Blvd, Tampa, FL 33606, USA University of Tampa Tampa United States of America; 3 Leibniz-Institut zur Analyse des Biodiversitätwandels, Museum Koenig, Adenauerallee 160, 53113 Bonn, Germany Leibniz-Institut zur Analyse des Biodiversitätwandels, Museum Koenig Bonn Germany

**Keywords:** DNA barcoding, haplotype network, Isopoda, Persian Gulf, Redescription, SEM

## Abstract

The woodlouse species *Tylosmaindroni* Giordani Soika, 1954 (Crustacea, Isopoda, Oniscidea) is redescribed from the Persian Gulf based on light and scanning electron microscopy. This species differs from the closely related *T.exiguus* Stebbing, 1910, from the Red Sea (coasts of Sudan and Eritrea), and Socotra Island, by pereopod 1 superior margin without a prominent projection and pleopod 2 endopod 2.3 times as long as exopod, vs. 3.6 in *T.exiguus*. A distribution map for *T.maindroni* is provided. In addition, we studied the molecular differentiation of five populations of *T.maindroni* from the Persian Gulf, based on partial *cytochrome c oxidase* subunit I (*COI*) gene sequences. The results revealed low levels of population structuring between the analyzed populations.

## ﻿Introduction

The isopod genus *Tylos* Audouin, 1826 has a worldwide distribution, with 21 species currently considered as valid (Boyko et al. 2008 onwards). Species in this genus are found in the marine sandy supralittoral zone, where animals can feed on algae and other organic material washed up on the beach by the waves (Kensley 1974). To avoid excessive predation by daytime predators (birds, crabs), feeding occurs at night (Schmalfuss and Vergara 2000). These animals are able to roll up into a perfect ball, with the antennae remaining inside the ball. Endoantennal conglobation can be also observed in Armadilliidae, Eubelidae, and Scleropactidae. Rolling up is not only a response to predators, but it can also help reduce water loss by about 35% (Schmalfuss and Vergara 2000; Sfenthourakis et al. 2020). According to Schmalfuss (2003), only two species of *Tylos* have been recorded from the northwestern areas of the Indian Ocean: *T.exiguus* Stebbing, 1910 from the Red Sea (coasts of Sudan and Eritrea) and the coasts of Socotra Island (Schmalfuss and Vergara 2000; Taiti and Ferrara 2004; Taiti and Checcucci 2010); and *T.maindroni* Giordani Soika, 1954 from the Gulf of Oman and the Persian Gulf. The original description of *T.maindroni* is brief and based on a single female from Muscat, Oman. Taiti and Ferrara (1991) later reported this species from Kuwait, Oman, and Iran (Busher coast), with illustrations of specimens from Kuwait. Recently, a molecular phylogenetic study including various species of the genus *Tylos* clearly revealed the existence of two distinct *Tylos* species along the coastal zones of the Arabian Peninsula (northwestern Indian Ocean): *T.maindroni* and *T.exiguus* (Hurtado et al. 2014).

Herein, we redescribe *T.maindroni* based on material from the Persian Gulf and provide new *COI* mtDNA sequence data.

## ﻿Materials and methods

### ﻿Morphological analyses

**Figure 1.**
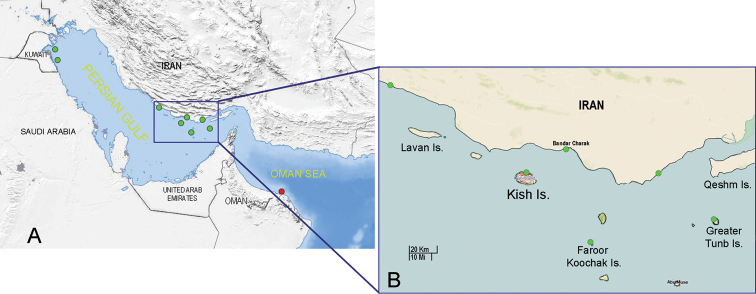
Map showing geographic distribution of *Tylosmaindroni* Giordani Soika, 1954 **A** the Persian Gulf and Gulf of Oman **B** the Persian Gulf. Green circles = in the Persian Gulf, Red circle = type locality.

Specimens used in this study were collected from four coastal sites in the Persian Gulf, Iran during field expeditions from 2006 to 2021 (Fig. 1; Table 1). All specimens are held in the isopod collection of the Zoological Museum of Shahrekord University (**ZMSU**).

**Table 1.** Samples of the *Tylosmaindroni* from the Persian Gulf used in this study.

Museum number	Voucher numbers	Coordinates	Collection date	Locality	GenBank Accession number COI
ZSMU 1206	1043	26°16'20.48"N, 55°17'28.00"E	03.01.2021	Greater Tunb Island	OK513061
ZSMU 1206	1044	26°16'20.48"N, 55°17'28.00"E	03.01.2021	Greater Tunb Island	OK513060
ZMSU 1205	1051	26°6'59.99"N, 54°26'10.88"E	30.12.2017	Faroor Koochak Island	OK513062
ZMSU 1201	1098	26°42‘555"N, 54°14‘329"E	05.12.2008	Bandar-e-Charak	OK513063
ZMSU 1201	C2	26°42‘555"N, 54°14‘329"E	05.12.2008	Bandar-e-Charak	OK513064
ZMSU 1202	B1	27°07‘113"N, 53°01‘418"E	30.01.2006	Banda-e Bostaneh	OK513065
ZMSU 1202	B2	27°07‘113"N, 53°01‘418"E	30.01.2006	Banda-e Bostaneh	OK513066

Specimens prepared for SEM were washed in a chilled 1% sodium acetate solution for 10 minutes, then cleaned for 10–20 seconds in an ultrasound cleaner in a weak solution of jewelry soap and distilled water to remove sediment and debris adhering to the cuticle. Specimens were dehydrated in an ethanol series (70, 75, 80, 85, 90, 95, 100%; 20 minutes per treatment). Specimens were transferred through ethanol and hexamethyldisilazane (**HMDS**) solutions (ethanol: HMDS ratios were 2:1, 1:1, 1:2) and finally into 100% HMDS (20 minutes per treatment). All samples were transferred to fresh HMDS, which evaporated overnight. Specimens were mounted on stubs using double adhesive carbon spots before being coated with gold in a sputter coater to 40 nm thickness. Micrographs were taken using a Hitachi S-2460N SEM at Zoologisches Forschungsmuseum Alexander Koenig in Bonn, Germany. Color images were taken using a Zeiss AxioCam ERc5s camera mounted on a Zeiss Stereomicroscope (Stemi 508).

### ﻿Molecular analyses

We extracted genomic DNA from the legs of seven specimens, 1–2 individuals per locality, using the Aron-Gene Tissue DNA Extraction kit (Aron-Gene, Iran) following the manufacturer’s protocol. A 536 base pair fragment of the mitochondrial *Cytochrome Oxidase I* (COI) gene was PCR-amplified using the LCO-1490 and HCO-2198 primer pair under standard conditions (Folmer et al. 1994). The PCR solution consisted of a 10 µl PCR Master Mix (SinaClon BioScience, Iran), 2 µl of template DNA (~50 ng), 1 µl of each primer (concentration 10 pm/ml), and 6 µl of nuclease-free water for a total volume of 20 µl. PCR products were examined using gel electrophoresis on 1% agarose gels, with positive PCR amplifications sequenced on an ABI 3130XL automated sequencer. We assembled, inspected, and edited sequences using Bioedit v.7.0.5.3.

Once assembled and edited, sequences produced in this study were combined with previously published COI sequences of *T.maindroni* as well as other *Tylos* species, provided that these sequences were > 500-bp long. Information for publicly available sequences included in this study can be found in Table 2. Sequences were aligned using the online MAFFT server (Katoh et al. 2019) and default settings. The resulting alignment was trimmed to remove end gaps. No evidence suggestive of pseudo-genes was observed in the final alignment. Given the high levels of divergence amongst *Tylos* species and differences in sequence lengths across studies, we re-aligned the COI sequences for *T.maindroni* individuals separately.

**Table 2.** GenBank Accession information for sequences used in this study. Accession numbers of sequences produced in this study are in bold.

	Number of individuals	GenBank Acc. No
*T.maindroni*	8	KJ468116; **OK513060 – OK513066**
*T.capensis*	33	MZ540108-MZ540140
*T.chilensis*	1	KJ468109
*T.exiguous*	1	KJ468112
*T.granulatus*	179	MK603245-MK603423
*T.granuliferus*	123	AB763432-AB763552; KJ468113-KJ468114
*T.marcuzzii*	1	KJ468118
*T.niveus*	1	KJ468120
*T.opercularis*	1	KJ468121
*T.punctatus*	23	KF007550-KF007555; KF007571-KF007574; KF007582-KF007586; KF007596-KF007598; KF007607-KF007608; KF007686-KF007688
*Tylossp.* BOLD:ACM2291	1	KJ592778
*Tylossp.* clade B^*^	1	KF007644
*Tylossp.* clade C^*^	1	KF007626
*Tylossp.* clade D^*^	1	KF007575
*Tylossp.* clade F^*^	2	KF007689-KF007690
*Tylossp.* clade G^*^	13	KF007576; KF007654; KF007657; KF007669-KF007671; KF007679-KF007680; KF007685; KF007698; KF007711-KF007712; KF007718
*Tylossp.* clade H^*^	9	KF007599; KF007609-KF007611; KF007615-KF007616; KF007646-KF007648
*Tylossp.* clade I^*^	7	KF007569; KF007664; KF007667-KF007668; KF007705; KF007713; KF007715
*Tylossp.* hachijoMN12	1	AB763553
*Tylossp.* outgroup^*^	1	KF007724
*T.spinulosus*	1	KJ468125
*T.wegeneri*	1	KJ468126

^*^ = Clades reported by Hurtado et al. (2014).

We used ASAP (Puillandre et al. 2021), a distance-based species delimitation approach, to determine if all *T.maindroni* sequences were assigned to a single species cluster. This analysis was carried out on the ASAP web portal (https://bioinfo.mnhn.fr/abi/public/asap/) under the Kimura (K80 or K2P) model (Kimura 1980) and a ts/tv ratio of 2.0. All other settings were as default. We estimated pairwise genetic distances with the Kimura-2-Parameter (K2P) correction in MEGA v11.0.10 (Tamura et al. 2021).

Lastly, we visualized relationships between *T.maindroni*COI haplotypes by reconstructing branch connections using the TCS network option (Clement et al. 2002) of PopArt v1.7 (Leigh and Bryant 2015), with a 95% connection limit.

## ﻿Systematic account

### ﻿Order Isopoda Latreille, 1817

**Suborder Oniscidea Latreille, 1802**

#### Family Tylidae Milne-Edwards, 1840

##### ﻿Genus Tylos

Taxon classificationAnimaliaIsopodaTylidae

Audouin, 1826

B4F4BF04-8D7D-5E00-A3E4-ACF33F561BD8

###### Type species.

*Tyloslatreillii* Audouin, 1826; from an unspecified location in Egypt (type locality); but current status is a nomen dubium (Taiti and Ferrara 1996: 460).

###### Diagnosis.

A diagnosis for the genus was published by Schmalfuss (2000).

##### ﻿ Tylos maindroni

Taxon classificationAnimaliaIsopodaTylidae

Giordani Soika, 1954

E48662E4-BC12-55FF-A4F8-F12EF9E9B5DA

**Figure 2.**
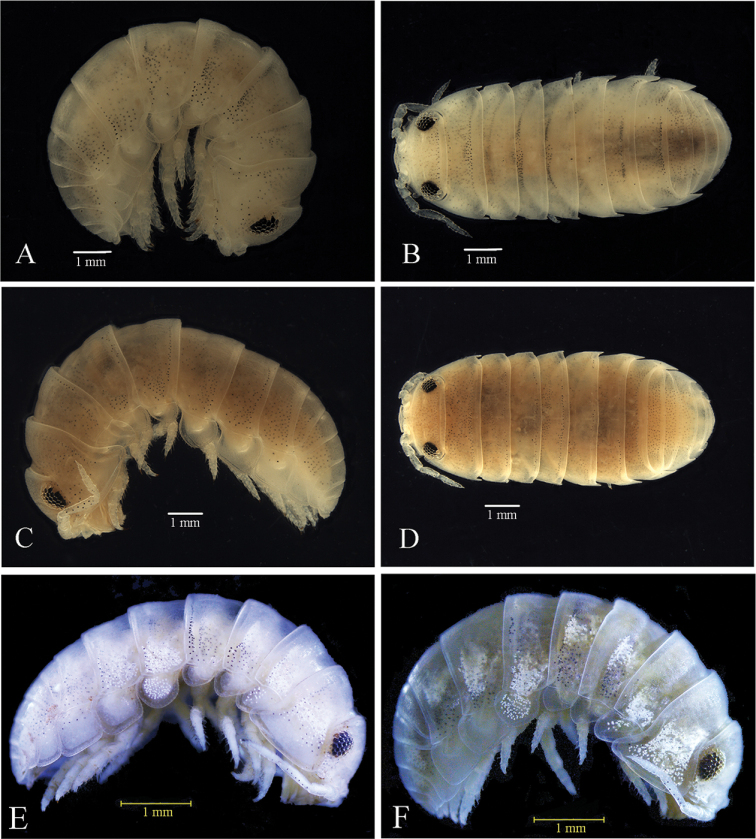
*Tylosmaindroni* Giordani Soika, 1954 **A, B** male from the Persian Gulf, Bandar-e-Charak (ZMSU 1201) **C, D** female from the Persian Gulf, Bandar-e-Charak (ZMSU 1201) **E** female from Faroor Koochak Island (ZMSU 1205) **F** male from Greater Tunb Island (ZSMU 1206).

**Figure 3.**
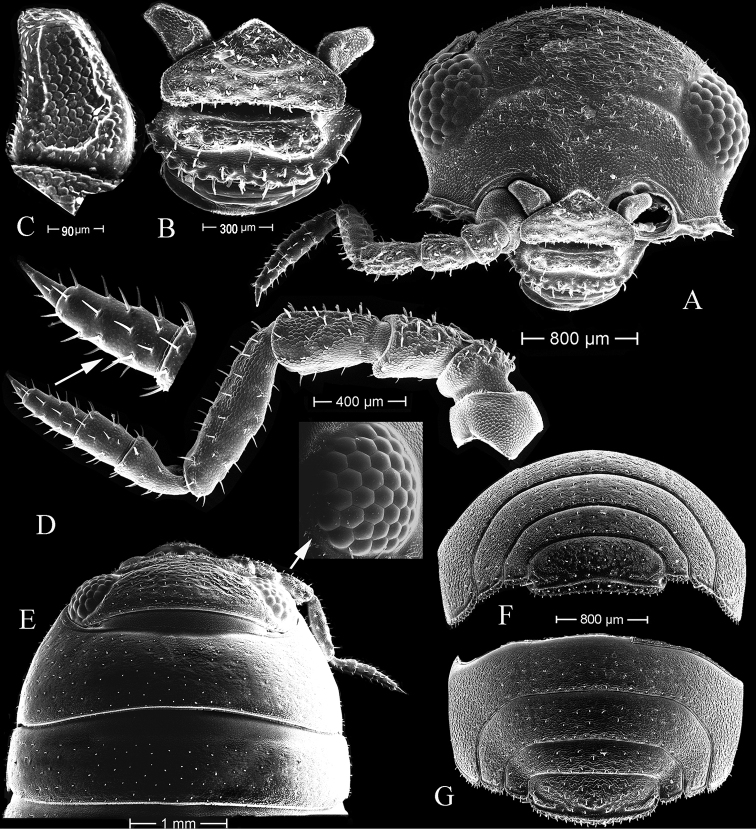
*Tylosmaindroni* Giordani Soika, 1954, male from the Persian Gulf, Bandar-e-Charak, scanning electron micrographs **A** head, frontal view **B** epistome **C** antennule **D** antenna **E** head, dorsal view **F** pleon and pleotelson, caudal view **G** pleon and pleotelson, dorsal view.

**Figure 4.**
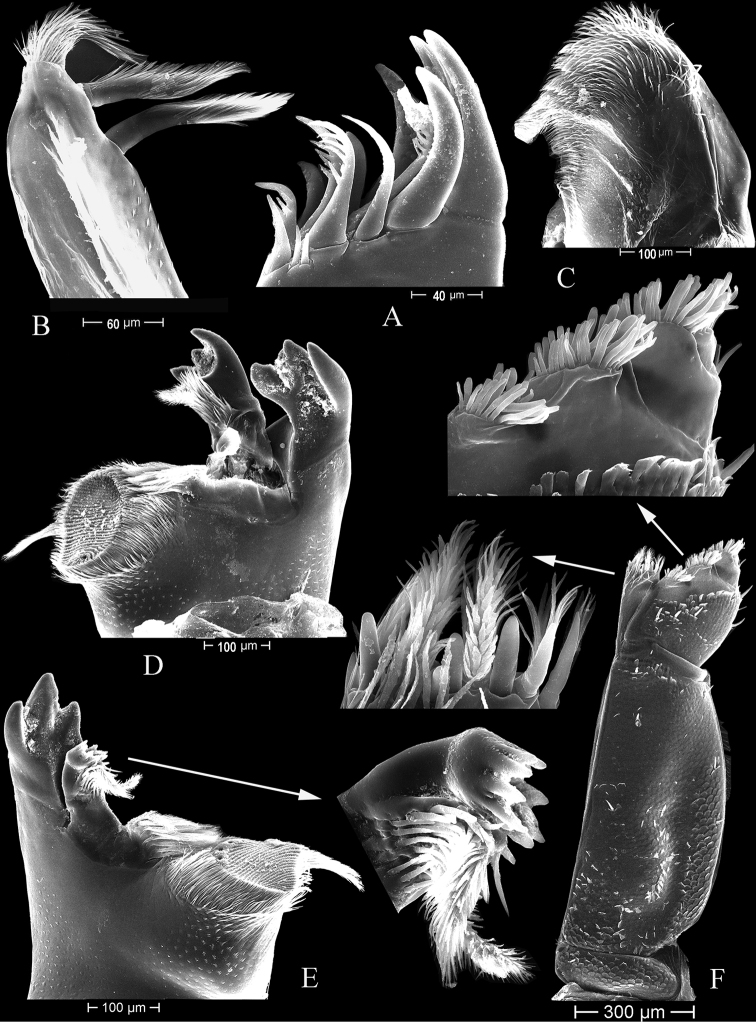
*Tylosmaindroni* Giordani Soika, 1954, male from the Persian Gulf, Bandar-e-Charak, scanning electron micrographs **A** maxillule, lateral endite **B** maxillule, medial endite **C** maxilla **D** left mandible **E** right mandible **F** maxilliped.

**Figure 5.**
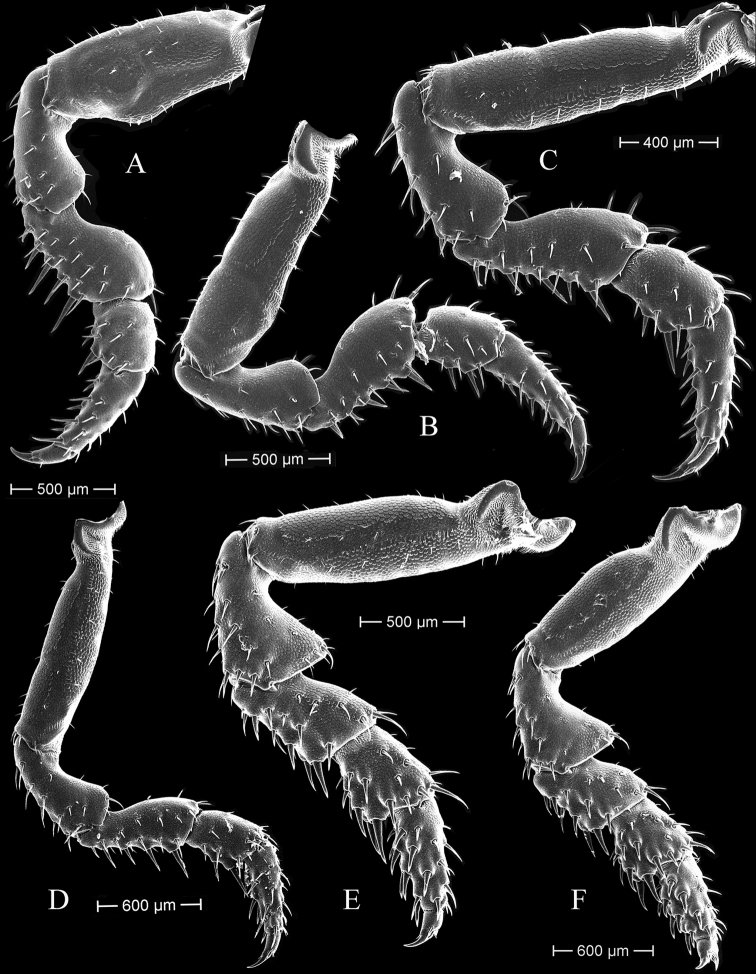
*Tylosmaindroni* Giordani Soika, 1954, male from the Persian Gulf, Bandar-e- Charak, scanning electron micrographs **A–F** pereopods 1–7, respectively.

**Figure 6.**
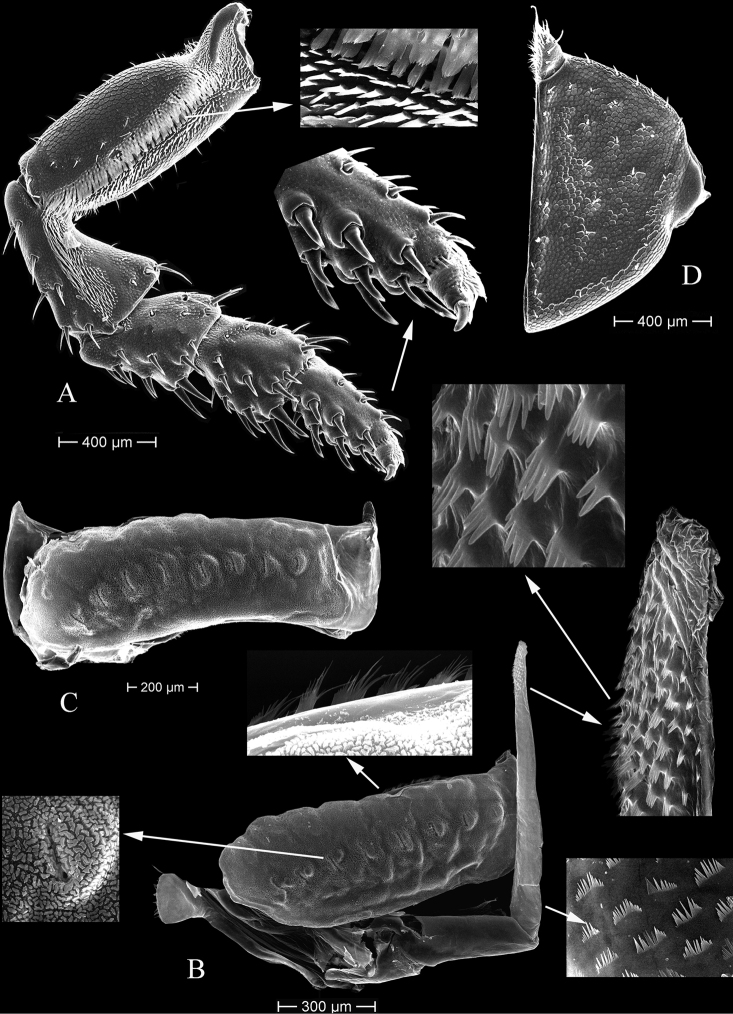
*Tylosmaindroni* Giordani Soika, 1954, male from the Persian Gulf, Bandar-e- Charak, scanning electron micrographs **A** pereopod 7 **B** pleopod 2 **C** pleopod 3 **D** uropod.

Figs 2 , 3 , 4 , 5 , 6

Tylos maindroni Giordani Soika, 1954: 76, figs 8, 9, pl. 10, Oman Sea, Muscat (type locality); Ferrara and Taiti 1986: 94; Taiti and Ferrara 1991: 213, fig. 3; Taiti et al. 2000: 148; Hurtado et al. 2014: 3, fig. 1.

###### Material examined.

7 ♂♂ (5.1 to 9.8 mm), 3 ♀♀ (5.5, 8.5, 10 mm), the Persian Gulf, Bandar-e-Charak, sandy shore, under wood block and rubbish on sand, 05 Dec. 2008, 26°42'555"N, 54°14'329"E, coll. V. Khalaji (ZMSU 1201); 8 ♂♂ (5 to 8 mm), 6 ♀♀ (6 to 9.2 mm), Bandar-e-Bostaneh, sandy shore, 03 Jan. 2006, 27°07'113"N, 53°01'418"E, coll. R. Naderloo (ZMSU 1202); 1 ♀ (12.2 mm), Bandar-e-Lengeh, sandy beach, beneath wood, 03 May 2010, 26°34'10"N, 54°54'21"E, coll. V. Khalaji (ZMSU 1203); 2 ♀♀ (9 and 10 mm), Kish Island, northern coast, Derakht-e-Sabz, 24 Jun. 2006, 26°34'102"N, 53°58'098"E, coll. V. Khalaji (ZMSU 1204); 3 ♂♂ (9 to 11mm); 8 ♀♀ (8 to 10 mm), Faroor Koochak Island, rocky, sandy western coast, 30 Dec. 2017, 26°65'999"N, 54°26'108"E, coll. V. Khalaji (ZMSU 1205); 10 ♀♀ (7 to 11mm), 5 ♂♂ (7.7 to 11 mm), Greater Tunb Island, sandy beach, 03 Jan. 2021, 26°16'20.48"N, 55°17'28.00"E, coll. V. Khalaji and M. Majidi (ZSMU 1206).

###### Redescription of male

**(from the Persian Gulf).** Color yellowish, or light brown dorsally with small, dark, pigmented dots of various densities (Fig. 2A–D), about 2.5 times as long as greatest width. ***Cephalon*** with a weak domed process on each side between eyes. ***Epistome*** triangular with narrowly rounded apex, labrum with rows of small tubercles, as figured (Fig. 3B). ***Eyes*** composed of 36–38 ommatidia in adults (Fig. 3E). Coxal plates 2–5 with rounded margin, coxal plates 6–7 rectangular with strait margin. ***Pleotelson*** framed by pleonite 5 laterally, distal margin with small setae, length about 0.55 times width (Fig. 3F, G).

***Antennula*** (Fig. 3C). Small, disolateral and apical margins straight, medial margin concave, covered with cuticular scales, about 1.3 times as long as greatest width.

***Antenna*** (Fig. 3D). Extending to posterior margin of pereonite 1, basal peduncular articles 2–5 increasing in length; article 5 about 1.3 times as long as article 4; flagellum with 4 articles, distal article smallest, apex with cone-like tuft of setae.

***Left mandible*** (Fig. 4D). Pars incisiva with three cusps; lacinia mobilis with three cusps and 2 penicils; pars molaris with flat grinding surface and with a tuft of numerous hair-like setae on margin.

***Right mandible*** (Fig. 4E). Pars incisiva with three cusps; lacinia mobilis with some small, sharp cusps (about 8) and 2 penicils; pars molaris grinding surface smaller than on left mandible, with a tuft of numerous hair-like setae on proximal margin.

***Maxillule*** (Fig. 4A, B). Lateral endite with 12 robust, simple or serrate setae; mesial endite with 2 subapical and 1 apical penicils.

***Maxilla*** (Fig. 4C). Apical margin round, densely setose.

***Maxilliped*** (Fig. 4F). Endite apical margin with 6 robust, simple setae, 3 large penicils, and 2 smaller penicils; palp of five articles, articles 2–5 each bearing a tuft of marginal, rod-like setae.

***Pereopod 1–7*** (Figs 5A–F, 6A). All with a rich armature of robust setae; pereopod 1 basis about 2.1 times as long as wide, ventral margin with a weak extension; pereopods 2–5 with longer basis; pereopod 7 (Fig. 6A) basis about 2.3 times as long as wide, with water conducting scale-rows.

***Pleopod 2*** (Fig. 6B). Exopod equipped with open lungs consisting of 8 pores, distal margin with cuticular scale. Endopod elongated, well extended beyond exopod distal margin, apical part bearing hand-like scales with 2–7 “fingers” that are directed proximally, proximal third covered with cuticular scales.

***Pleopod 3*** (Fig. 6C). Exopod equipped with open lungs consisting of 9 pores.

***Uropod*** (Fig. 6D). Protopod (peduncle) with straight medial margin, disto-lateral margin with 6 small marginal setae, length about 1.62 greatest width; exopod small, about 0.23 times length of protopod, covered with small setae medially.

**Female** (Fig. 2C, D). Apart from sexual characters, similar to male.

###### Distribution.

Oman, the Persian Gulf (Kuwait; Bandar-e-Charak, Bandar-e-Bostaneh, Bandar-e-Lengeh, Kish, Greater Tunb, and Faroor Koochak Islands, Iran)

## ﻿Results

### ﻿Genetic differentiation

**Figure 7.**
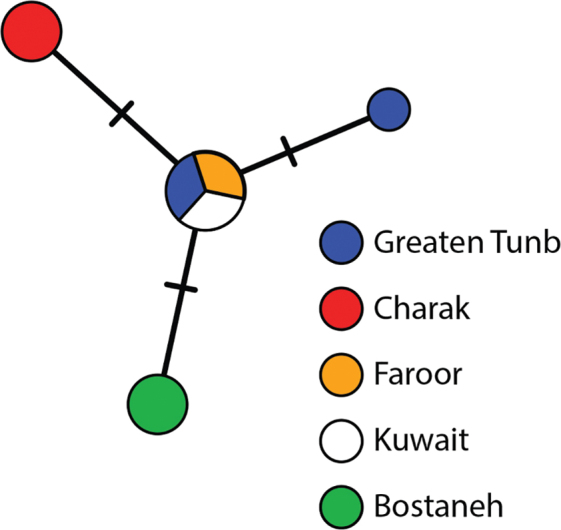
Haplotype networks for the COI mitochondrial gene fragment of *Tylos* from the Persian Gulf. Colors correspond to locations as indicated in figure. Dashes along branches represent the number of nucleotide differences between haplotypes. Frequency of haplotype recovery represented through relative sizes of circles.

We obtained seven 534-bp long *COI* sequences from *T.maindroni* individuals from four locations across the Persian Gulf coastline of Iran. These sequences were combined with a previously published *COI* sequence of *T.maindroni* from Kuwait (GenBank Acc. KJ468116; BIN: BOLD: ACQ3230). We identified four highly similar COI haplotypes as indicated by K2P divergences (0.0–0.4%, 1–3 nucleotide differences, Fig. 7). These haplotypes, however, were highly divergent from those found in other *Tylos* species (16.2–33.9% COIK2P divergences, Table 3).

**Table 3.** Average COIK2P divergences amongst *Tylos* species included in this study.

	*T.maindroni*	*T.punctatus*	*Tylossp.* BOLD:ACM2291	*Tylossp.* clade F^*^	*Tylossp.* clade D^*^	*Tylossp.* clade G^*^	*Tylossp.* clade H^*^	*Tylossp.* clade I^*^	*Tylossp.* clade C^*^	*Tylossp.* clade B^*^	*Tylossp.* outgroup^*^	*T.niveus*	*T.granulatus*	*T.capensis*	*T.marcuzzii*	*T.exiguus*	*T.opercularis*	*T.chilensis*	*T.spinulosus*	*Tylossp.* hachijoMN12	*T.granuliferus*	*T.wegeneri*
*T.maindroni*	< 0.5																					
*T.punctatus*	20.5	< 5.8																				
*Tylossp.* BOLD: ACM2291	20.5	0.1	N/A																			
*Tylossp.* clade F^*^	19.2	14.9	14.8	0.0																		
*Tylossp.* clade D^*^	17.7	12.5	12.4	11.0	N/A																	
*Tylossp.* clade G^*^	18.2	13.6	13.6	13.2	12.5	< 6.2																
*Tylossp.* clade H^*^	18.6	14.0	14.1	12.6	12.2	4.6	0.0															
*Tylossp.* clade I^*^	18.9	14.2	14.3	13.6	13.0	5.6	4.5	0.0														
*Tylossp.* clade C^*^	21.2	15.6	15.5	12.1	14.2	13.5	14.0	13.9	N/A													
*Tylossp.* clade B^*^	20.4	12.9	12.8	14.5	13.1	13.4	13.1	13.4	13.1	N/A												
*Tylossp.* outgroup^*^	19.9	16.0	15.9	16.7	18.2	15.8	15.9	16.3	18.4	14.7	N/A											
*T.niveus*	17.7	16.0	15.8	15.1	16.8	15.5	17.1	16.8	16.6	15.9	15.6	N/A										
*T.granulatus*	17.4	15.9	15.9	15.7	15.0	15.2	15.9	15.8	18.5	16.7	19.1	15.8	< 13.2									
*T.capensis*	19.5	16.3	16.3	17.9	17.7	17.5	18.4	17.7	20.8	17.8	19.0	19.8	12.2	< 2.8								
*T.marcuzzii*	21.5	17.8	17.8	21.6	22.7	20.2	20.9	21.3	19.9	17.3	18.0	20.6	19.4	19.6	N/A							
*T.exiguus*	20.7	21.3	21.2	20.5	19.2	18.7	18.8	19.2	21.9	20.5	21.0	22.9	18.9	19.1	23.1	N/A						
*T.opercularis*	25.4	25.5	25.5	21.9	25.2	25.1	25.2	25.2	27.3	25.8	23.6	19.2	23.3	23.3	26.0	23.0	N/A					
*T.chilensis*	25.6	23.7	23.6	26.2	25.4	24.9	25.0	24.6	24.8	25.1	24.3	25.5	22.7	24.8	24.4	24.1	29.1	N/A				
*T.spinulosus*	23.2	21.1	21.0	22.6	22.2	21.0	21.4	22.7	22.8	21.3	23.5	23.0	20.9	22.1	27.0	22.0	31.3	13.7	N/A			
*Tylossp.* hachijoMN12	23.9	27.3	27.3	25.1	26.6	25.2	27.4	27.0	25.7	24.9	26.2	21.2	25.2	25.8	29.7	28.2	24.1	33.5	28.3	N/A		
*T.granuliferus*	28.9	30.5	30.4	27.1	25.6	27.6	27.2	27.2	28.0	27.5	31.5	27.3	25.6	26.2	32.0	26.3	24.4	32.2	30.3	24.4	< 25.2	
*T.wegeneri*	26.2	28.7	28.7	27.3	27.2	26.3	25.4	27.7	26.2	26.0	27.7	27.4	25.9	26.0	25.7	27.1	26.4	27.8	27.3	26.5	29.2	N/A

^*^ = Clades reported by Hurtado et al. (2014).

Combining the *T.maindroni* sequences with other previously published sequences of the genus *Tylos* resulted in a 517-bp long alignment containing 410 sequences. ASAP analyses of this dataset identified two partitioning schemes with nearly similar numbers of hypothetical species groups (23 and 24), threshold distances (0.068107 and 0.051440), and low ASAP scores (7 and 8). This last measure reflects both the p-value and the relative barcode gap width rank for a given partitioning scheme, with lower values reflecting stronger support for a given partitioning scheme. All COI haplotypes from *T.maindroni* individuals were placed in a single cluster that included no sequences from other *Tylos* species, regardless of the partitioning scheme.

## ﻿Discussion

*Tylosmaindroni* was first described by Giordani Soika in 1954; however, the original description was brief and did not include a discussion or illustration of characters used in the taxonomy of this genus. A later work by Taiti and Ferrara (1991) suggested that *T.maindroni*’s geographic range extends into the Persian Gulf, including locations on the coasts of Kuwait and Iran, but additional work remains necessary to clarify the status of this species and its geographic range. Additionally, considering the high levels of genetic divergence reported in several coastal isopod taxa (Hurtado et al. 2013; Khalaji-Pirbalouty and Raupach 2014, 2016; Raupach et al. 2014; Hurtado et al. 2017; Santamaria et al. 2017; Greenan et al. 2018; Hurtado et al. 2018; Santamaria 2019), it would be important to determine if *T.maindroni* harbors cryptic diversity in its native range.

Our Persian Gulf specimens correspond morphologically quite well to the brief description and illustrations of *T.maindroni* from Oman by Giordani Soika (1954) and from Kuwait by Taiti and Ferrara (1991). Nevertheless, there is a slight difference in the number of lung pores on the exopod of the pleopods: the exopod of pleopod 2 has 8 pores rather than 7 and the exopod of pleopod 3 has 9 pores rather than 8. *Tylosmaindroni* is morphologically most similar to *T.exiguus* Stebbing, 1910, a Red Sea species shown by Hurtado et al. (2014) to be a sister taxon to *T.maindroni* based on several mitochondrial markers. The former species differs from *T.maindroni* by having pereonite 1 posterior margin with a distinctly deeper concavity at the lateral side, pereopod 1 superior margin with a prominent projection, and pleopod 2 endopod 3.6 times as long as exopod (vs. 2.3 times in *T.maindroni*).

Molecular data are in concordance with the above findings. All *Tylos* specimens that were morphologically identified as *T.maindroni* have highly similar COI haplotypes differing by a maximum of three positions (K2P distances amongst haplotypes < 0.5%). Furthermore, sequences recovered from *T.maindroni* individuals were highly divergent from all other COI sequences recovered from other *Tylos* species including *T.exiguus* (16.2–33.9% COIK2P). Not surprisingly, all *T.maindroni* haplotypes were assigned to a single species cluster in species delimitation analyses carried out in ASAP, regardless of the partitioning scheme.

The low level of diversification herein reported between individuals of *T.maindroni* collected at Persian Gulf locations stands in contrast with those reported for other coastal oniscid taxa (Khalaji-Pirbalouty and Raupach 2014, 2016; Raupach et al. 2014; Hurtado et al. 2017; Santamaria et al. 2017; Greenan et al. 2018; Hurtado et al. 2018; Santamaria 2019), including other *Tylos* species (Hurtado et al. 2013). For instance, the molecular characterizations of *Tylos* populations from the Gulf of California showed genetic differentiation in COI sequences ranging from 3.6 to 17.3%, indicating long-standing isolation of the populations in the region as well as the possible presence of cryptic species (Hurtado et al. 2013). Similarly, *T.granulatus* populations in South Africa have shown to harbor two highly divergent mitochondrial lineages (Mbongwa et al. 2019). In contrast to this, the COIK2P divergences observed in *T.maindroni* were less than 0.5%.

The low levels of genetic divergence within *T.maindroni* in the Persian Gulf is likely a reflection of the young age of this marine waterbody. Although there is disagreement on the extent of the Persian Gulf coastline during the Holocene and Late Pleistocene (Sissakian et al. 2020), the Gulf Basin is thought to have been free of marine influence up until the last glacial maximum ~18,000 ya., with marine flooding due to rising sea levels and glacial displacement starting ~14,000 ya (Lambeck 1996). Thus, the geology of the region suggests that the ancestor to *T.maindroni* populations in the Persian Gulf area invaded the Gulf in the past ~14,000 years. Alternatively, the low divergence levels between COI sequences reported herein may be the result of infection with *Wolbachia*. Infection with this endosymbiotic bacterium has been proposed to reduce mitochondrial polymorphisms in arthropods, including isopods (Marcadé et al. 1999; Xiao et al. 2012; Delhoumi et al. 2019; but see Tang et al. 2019). We cannot determine whether *Wolbachia* have reduced mitochondrial diversity in *T.maindroni* in the Persian Gulf as we did not test for the presence of *Wolbachia* in our specimens. However, given the recent geological and hydrological history of the Persian Gulf, we propose that the low levels of divergence in *T.maindroni* reported herein are likely the result of the young age of the modern Persian Gulf. Nevertheless, future work remains needed to conclusively discern between these explanations. Future studies also remain needed to clarify the origins and evolution of *T.maindroni* in the region. The closest extant relative of *T.maindroni* in the Persian Gulf is *T.exiguus* (Hurtado et al. 2014), suggesting that the Persian Gulf populations of *T.maindroni* likely originated from an ancestor inhabiting coastal habitats in the Indian Ocean basin. As our sampling did not include *T.maindroni* populations from the Indian Ocean, future work would be best served by incorporating these populations.

## Supplementary Material

XML Treatment for Tylos

XML Treatment for Tylos maindroni
